# Supplementary material for: Iterative improvement in the automatic modular design of robot swarms
Source: PeerJ Comput Sci. 2020 Dec 7;6:e322. doi: 10.7717/peerj-cs.322 (PMC7924708; doi:10.7717/peerj-cs.322)
Supplement: Supplemental Information 3 [file peerj-cs-06-322-s003.zip › argos3/doc/api/standalone/a00632_source.html]

ARGoS: plugins/simulator/visualizations/qt-opengl/models/qtopengl\_cylinder.h Source File


- Main Page
- Related Pages
- Namespaces
- Classes
- Files

- File List
- File Members

# plugins/simulator/visualizations/qt-opengl/models/qtopengl\_cylinder.h

Go to the documentation of this file.

```
00001 
00007 #ifndef QTOPENGL_CYLINDER_H
00008 #define QTOPENGL_CYLINDER_H
00009 
00010 namespace argos {
00011    class CQTOpenGLCylinder;
00012    class CCylinderEntity;
00013 }
00014 
00015 
00016 #ifdef __APPLE__
00017 #include <gl.h>
00018 #else
00019 #include <GL/gl.h>
00020 #endif
00021 
00022 namespace argos {
00023 
00024    class CQTOpenGLCylinder {
00025 
00026    public:
00027 
00028       CQTOpenGLCylinder();
00029 
00030       virtual ~CQTOpenGLCylinder();
00031 
00032       void DrawLEDs(CCylinderEntity& c_entity);
00033       virtual void Draw(CCylinderEntity& c_entity);
00034 
00035    private:
00036 
00037       void MakeBody();
00038       void MakeLED();
00039 
00040    private:
00041 
00042       GLuint m_unBaseList;
00043       GLuint m_unBodyList;
00044       GLuint m_unLEDList;
00045       GLuint m_unVertices;
00046 
00047    };
00048 
00049 }
00050 
00051 #endif
```

---

Generated on 10 Jul 2018 for ARGoS by 
 1.6.1
